# Gab2 facilitates epithelial-to-mesenchymal transition via the MEK/ERK/MMP signaling in colorectal cancer

**DOI:** 10.1186/s13046-015-0280-0

**Published:** 2016-01-12

**Authors:** Chenbo Ding, Junmin Luo, Longmei Li, Shanshan Li, Liwen Yang, Hongfei Pan, Qianyi Liu, Huan Qin, Chao Chen, Jihong Feng

**Affiliations:** Department of Immunology, Zunyi Medical College, Immunology Innovation Base of Postgraduate Education in Guizhou Province, Zunyi, 563003 PR China; Department of Microbiology, Zunyi Medical College, Zunyi, 563003 PR China; Department of Oncology, the First Affiliated Hospital of Zunyi Medical College, Zunyi, 563003 PR China

**Keywords:** Gab2, Colorectal cancer, Metastasis, EMT

## Abstract

**Background:**

Grb2-associated binder 2 (Gab2), a scaffolding adaptor protein, has recently been implicated in cancer progression. However, the role of Gab2 in the progression and metastasis of colorectal cancer (CRC) remains unclear.

**Methods:**

Gab2 expression was assessed in CRC patient specimens as well as in CRC cell lines. Recombinant lentivirus vector containing Gab2 gene and its small interfering RNAs were constructed and introduced into CRC cells. Cell migration and invasion ability were evaluated by transwell assays in vitro, and in vivo metastasis was performed on nude mice model. Moreover, the expression of Gab2 and epithelial-to-mesenchymal transition (EMT)-associated proteins (E-cadherin and vimentin) were assessed by western blot and qRT-PCR in CRC cells to evaluate the correlation between Gab2 and EMT. Finally, we evaluated the impact of Gab2 on the activation of its downstream signaling effectors, and furthermore the effects of these pathways on Gab2 induced-EMT were also detected.

**Results:**

We confirmed that increased Gab2 expression correlated with higher tumor node metastasis stage and highly invasive CRC cell lines. Ectopic expression of Gab2 promoted metastasis of CRC cells, whereas silencing of Gab2 resulted in inhibited metastasis both in vitro and in vivo. Overexpression of Gab2 in CRC cells induced EMT, whereas knockdown of Gab2 had the opposite effect. Furthermore, upregulation of Gab2 expression obviously stimulated the activation of extracellular signal-regulated kinase-1/2 (ERK1/2), and increased the expression of matrix metalloproteinase-7 (MMP7) and matrix metalloproteinase-9 (MMP9) in CRC cells. Conversely, downregulation of Gab2 expression significantly decreased the activation of ERK1/2, and inhibited MMP7 and MMP9 expression. U0126, an inhibitor of mitogen-activated protein kinase (MEK), can reverse the effects of Gab2 on EMT.

**Conclusions:**

Our work highlights that Gab2 induces EMT through the MEK/ERK/MMP pathway, which in turn promotes intestinal tumor metastasis.

**Electronic supplementary material:**

The online version of this article (doi:10.1186/s13046-015-0280-0) contains supplementary material, which is available to authorized users.

## Background

Colorectal cancer (CRC) is the third most commonly carcinomas in males and the second in females throughout the world, with an estimated 1.4 million new cancer cases and nearly 0.7 million deaths each year [1]. The CRC incidence in the People’s Republic of China has been increasing annually and it will continue to rise in the next years [2]. Although the survival rate for patients with CRC has increased at early stages, as a result of improved detection and increased awareness, the long-term survival rate still remains very poor, mainly due to local recurrence and distant metastases formation [3, 4]. Metastasis, one of the six initial cancer hallmarks [5], which is a main reason of CRC-associated survival rate depressed [6]. Approximately 35 % of patients with CRC have metastatic disease at diagnosis and more than one-third of patients will ultimately develop metastatic disease, however, the exact molecular mechanism underlying CRC metastasis is little known [7–9]. Improving understanding the key molecules in these processes, may provide novel insight for designing effective anti-cancer therapies.

Grb2-associated binder 2 (Gab2), a member of the DOS/Gab family of scaffolding adapters, has been reported to play important roles in the progression and metastasis of human cancers, particularly in breast and ovarian cancers and melanoma [10]. Although Gab2 has a modest impact on mammary tumor initiation/growth, Gab2 deletion leads to impaired ERK activity and attenuated mammary tumor metastasis [11]. Gab2 triggers epithelial-to-mesenchymal transition (EMT) and promotes the migration and invasion of ovarian cancer cells through activation of the phosphatidylinositol 3-kinase (PI3K) pathway [12]. In melanoma, Gab2 expression promotes the migration, invasion and metastasis of tumor cells via activation of the PI3K/AKT signaling [13]. These data indicates that Gab2 and Gab2-mediated signaling pathways are involved in the metastasis of human tumor cells.

However, whether Gab2 has any role in the metastasis of CRC and its underlying mechanism remains unknown. In this study, the effects of Gab2 on CRC metastasis as well as its relative molecular mechanism were investigated using in vitro CRC cell lines and in vivo mouse model systems. We demonstrate that Gab2 induces EMT by the MEK/ERK/MMP pathway, which in turn promotes CRC cells metastasis. The current findings suggest that Gab2 plays a vital role in regulating CRC metastasis and may serve as a potential target for diagnosis and therapy in CRC.

## Methods

### Tissue samples

Samples from 35 CRC patients who had undergone proctocolectomy with lymph node (LN) dissection for CRC at the Department of gastrointestinal Surgery, the First Affiliated Hospital of Zunyi Medical College (Zun’yi, China) between May 2015 and July 2015 were included in the study. Patients did not receive neoadjuvant therapy. After surgical resection, the resected specimens were histologically examined by hematoxylin and eosin (HE)-staining and were immediately put into liquid nitrogen until further use. Total RNA from the frozen tissues was isolated with Trizol (Invitrogen, USA) according to the manufacturer’s instructions. All patients provided written informed consent before surgery, and our study were approved by the Ethics Committee of the First Affiliated Hospital of Zunyi Medical College according to the 1975 Declaration of Helsinki.

### Cell lines and culture conditions

The human CRC cell lines HT29, SW480, SW620 and LOVO, and a normal human intestinal epithelial cell line FHC were obtained from the American Type Culture Collection (ATCC, Manassas, USA), and authenticated according to the ATCC recommendations. SW480 and SW620 cells were cultured in Leibovitz’s L-15 medium (GIBCO Laboratories, Grand Island, NY, USA) supplemented with 10 % fetal bovine serum (FBS) (HyClone, Logan, UT, USA), 100 U/ml penicillin and 100 μg/ml streptomycin. HT29 cells were cultured in McCoy’s medium (GIBCO) supplemented with 10 % FBS and antibiotics. LOVO and FHC were maintained in RPMI-1640 medium (HyClone) supplemented with 10 % FBS and antibiotics. All the cells were cultured at 37 °C in a humidified incubator of 5 % CO_2_.

### RNA extraction and real-time PCR

Total RNA was extracted using Trizol (Invitrogen, USA) according to the manufacturer’s instructions. The obtained RNA was first reversely transcribed into cDNA by using RT reagent Kit (TakaRa, Japan). Quantitative reverse transcription-PCR (qRT-PCR) analysis was performed as previously described [14]. The sequences of primers in this section are the followings: (1) Gab2: 5′-GTGGGGGATCTGAATGTTTTTATG-3′ (forward) and 5′-GCCCCAGGGTAGAATGAAACG-3′ (reverse); (2) E-cadherin: 5′-GCTCGGCCTGAAGTGACTCG-3′ (forward) and 5′-CCGCTTCCTTCATAGTCAAACAC-3′ (reverse); (3) Vimentin: 5′-CCAGGCAAAGCAGGAGTCCAC-3′ (forward) and 5′-GCTTCCTGTAGGTGGCAATCTC-3′ (reverse); (4) GAPDH: 5′-GAAGGTGAAGGTCGGAGTC-3′ (forward) and 5′-GAAGATGGTGATGGGATTTC-3′ (reverse). GAPDH was used as an internal control.

### Western blot analysis

Western blot analysis was performed as previously described [14]. The following commercial antibodies were used in this study: Gab2 (OriGene Technologies, USA), E-cadherin, vimentin, MMP7 and MMP9 (Abcam, UK), phospho-AKT, total AKT, phospho-ERK1/2 and total ERK1/2 (Invitrogen, USA), GAPDH and β-actin (Immunology Consultants Laboratory, USA).

### Immunohistochemical staining

Immunohistochemical assay was performed on paraformaldehyde-fixed paraffin sections as previous reported [14]. The Gab2 (OriGene Technologies, USA) primary antibody was used at a 1:150 dilution in the immunohistochemistry assays. The immunostaining intensity and average percentage of positive cells were evaluated as previous reported [14]. According to the chromatosis intensity, no staining, light yellow, buffy and brown are scored 0, 1, 2, and 3, respectively. And the percentage of positive cells was categorized as the following grades: 0 (less than 5 %), 1 (5–25 %), 2 (26–50 %), and 3 (>51 %) accordingly. By multiplying the staining intensity and the percentage of positive cells, the final weighed expression score was ranged from 0 to 9. The sum of the staining intensity score and the percentage score was used to define the Gab2 protein expression levels: 0–2, low expression and 3–9, high expression.

### Lentivirus production and construct design

Retroviral expression vector CMV-puro and CMV-puro containing Gab2 gene were designed and provided by Cyagen Biosciences Inc. (Guangzhou, China). On the basis of the Gab2 sequence, one short hairpin RNA was designed using the small interfering RNAs (siRNAs) Target Finder (InvivoGene, San Diego, CA, USA). The effective Gab2 siRNA sequence is 5′- GCACCAATTCTGAAGACAA -3′, and the control-siRNA sequence is 5′-TTCTCCGAACGTGTCACGT-3′. Lentiviral vectors encoding siRNAs and the control-siRNA sequence were designed and provided by Genechem lnc. (Shanghai, China).

### Migration and invasion assay

Migration and invasion assays were using Transwell system (24-well, 8 μm pore size with polycarbonate membrane; Corning Costar, Lowell, MA, USA). Six hundred microlitres of the medium with 15 % FBS was added to the lower well of each chamber and 5 × 10^4^ cells/well resuspended in 200 μl serum-free medium were added to the upper inserts. After 48 h incubation, the cells that had migrated through the filter into the lower wells were stained with 0.1 % crystal violet and counted using fluorescence microscopy. The invasion assay was performed in the same way as the migration assay except that the membrane was coated with Matrigel (BD Biosciences, SanJose, CA, USA). Three independent experiments were performed with triplicate wells. DMSO (vehicle) or U0126 was added to both the top and bottom of transwell.

### *In vivo* metastasis development assay

To produce experimental lung and liver metastasis, SW480-NC, SW480-Gab2, SW620-si-Ctrl and SW620-Gab2si cells (1 × 10^6^ cells) were injected into the lateral tail veins of 5–6 weeks-old BALB/c nu/nu female mice (six mice per group). After 4 weeks, all the mice were killed under anesthesia. The lungs and livers were collected and fixed in 10 % formalin. For tissue morphology evaluation, HE-staining was performed on sections from embedded samples. All animal experiments were performed with the approval of Zunyi Medical College Animal Care and Use Committee.

### Statistical analysis

All values were represented as the mean ± SEM from at least three independent experiments. Pearson’s χ^2^-test was used for clinical correlative studies. Student’s t-test for two groups or one-way analysis of variance (ANOVA) for three or more groups were performed to evaluate the statistical significance. Differences were considered significant at *P* values less than 0.05.

## Results

### Gab2 is significantly upregulated in LN metastasis-positive CRC tissues

**Fig. 1:**
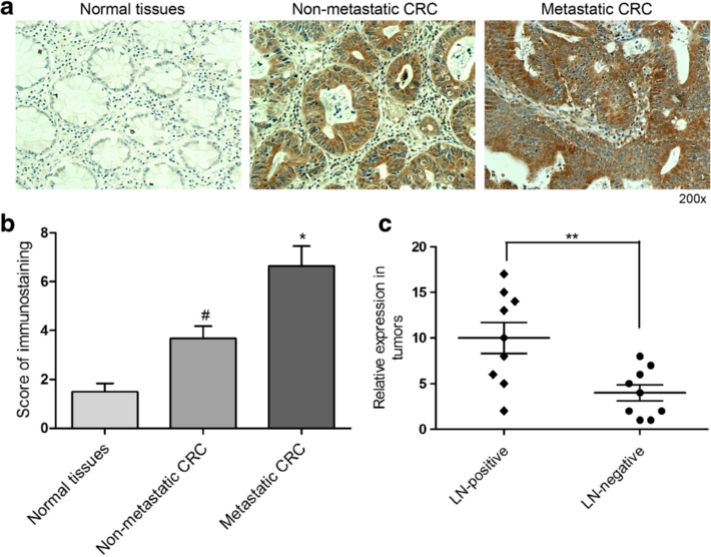
Gab2 is significantly upregulated in LN metastasis-positive CRC tissues. **a** Immunohistochemistry analysis of Gab2 expression in 35 paired CRC tissues. **b** Results of immunohistochemical staining were evaluated by the staining scores. *P < 0.05 vs non-metastatic CRC or normal tissues. ^#^ *P* < 0.05 vs normal tissues. **c** qRT-PCR analysis of Gab2 expression in human CRC primary tumors, LN-positive (*n* = 9) or -negative (*n* = 9). Matching normal colorectal tissue sample from the same patient was used for normalization

Our previous study has shown that Gab2 is overexpressed in CRC tissues, and this overexpression is significantly correlated with lymph node (LN) metastasis [14]. In this study, we also assessed Gab2 expression in a tissue microarray of 35 CRC patients (Additional file 1: Table S1). The results of immunohistochemical staining showed that Gab2 was significantly upregulated in primary sites of metastatic CRC compared with either non-metastatic CRC or normal tissues (Fig. 1a, b). To investigate the correlation between Gab2 overexpression and CRC metastasis, we detected Gab2 expression in 9 pairs of LN metastasis-positive (LN-positive group) and LN metastasis-negative (LN-negative group) primary CRC specimens. Real-time PCR analysis showed that Gab2 mRNA level was obviously higher in the LN-positive group than in the LN-negative group (Fig. 1c). Taken together, these results suggest that the expression of Gab2 is positively correlated with the metastasis of CRC.

### Gab2 accelerates CRC cell migration and invasion *in vitro*

**Fig. 2:**
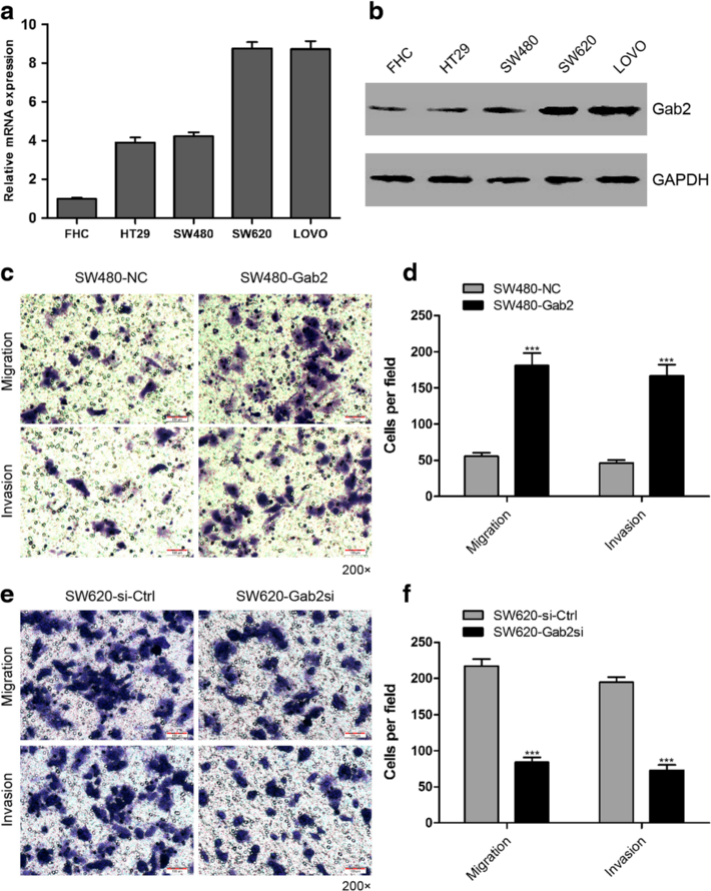
Gab2 promotes CRC cells migration and invasion in vitro. **a** Expression of Gab2 in four human CRC cell lines relative to the normal human intestinal epithelial cell line FHC was detected by qRT-PCR. **b** Western blot analysis of Gab2 expression in different CRC cell lines. **c** Migration and invasion assay of SW480-NC and SW480-Gab2 cells. **d** Migration and invasion of SW480-NC and SW480-Gab2 cells were quantitatively analyzed. Columns are the average of three independent experiments ± SEM. **e** and **f** Migration and invasion assay of SW620-si-Ctrl and SW620-Gab2si cells (two clones, **c** and **d**). ***P < 0.001

To determine whether Gab2 expression associates with the metastatic potential of CRC cells, we detected the expression of Gab2 in four human CRC cell lines (HT29, SW480, SW620 and LOVO) and in a normal human intestinal epithelial cell line FHC. The levels of Gab2 expression were obviously increased in SW620 and LOVO cells, which have highly metastatic abilities, compared with either the poorly metastatic cell lines HT29 and SW480 or the normal human intestinal epithelial cell line FHC (Fig. 2a, b). Considered that SW480 and SW620 cells were isolated from a same patient [15], these cells therefore have the same genetic background but different metastatic potential [16].

To explore whether Gab2 affects the metastatic phenotype of CRC cells, SW480 cells were infected with lentiviral vectors containing Gab2 gene or a control lentivirus and stable clones were established (SW480-Gab2 and SW480-NC, respectively). Gab2-specific small interfering RNAs (siRNAs) or its corresponding control siRNA were introduced into SW620 cells and stable clones were established (SW620-Gab2si and SW620-si-Ctrl, respectively). In order to determine the generality of the impact of Gab2 regulation in cell metastasis, we adopted transwell assays. As results, upregulation of Gab2 expression in SW480 cells significantly enhanced cell migration and invasion (Fig. 2c, d). Conversely, downregulation of Gab2 in SW620 cells markedly reduced cell migration and invasion (Fig. 2e, f). These data indicates that Gab2 promotes metastasis of CRC cells in culture.

### Gab2 induces metastasis in a xenograft model

**Fig. 3:**
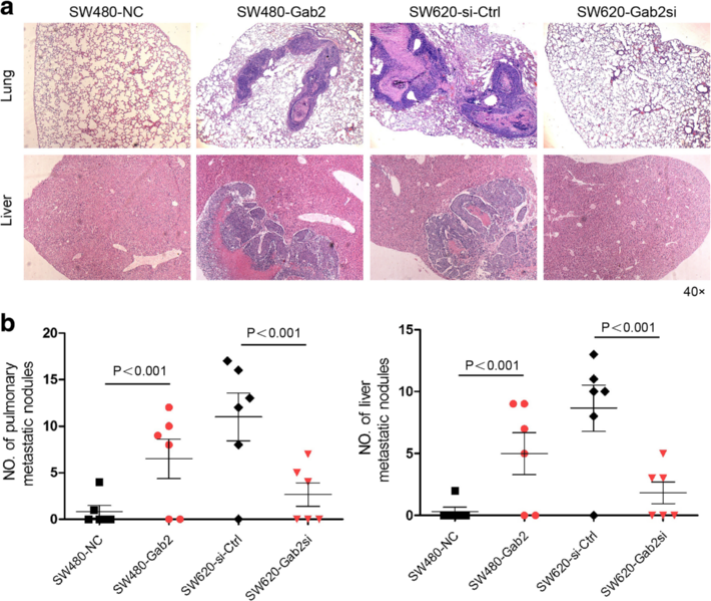
Gab2 enhances CRC cells metastasis in a xenograft model. **a** Images showing representative HE-staining of lungs and livers isolated from mice that received tail vein injection of SW480-NC, SW480-Gab2, SW620-si-Ctrl and SW620-Gab2si cells. Each group contains six mice. **b** The numbers of pulmonary and liver metastatic nodules were counted and analyzed with Student’s t-test

Next, we studied the effect of Gab2 on tumor metastasis in vivo. SW480-NC, SW480-Gab2, SW620-si-Ctrl and SW620-Gab2si cells were transplanted into the nude mice via tail vein injection. After 4 weeks, mice were anesthetized, and their lungs and livers were dissected and HE-staining was performed to evaluate tissue morphology. Histologic analyses confirmed that the incidence of lung and liver metastases was obviously enhanced in mice injected with SW480-Gab2 cells and significantly decreased in mice injected with SW620-Gab2si cells, compared with the corresponding control groups (Fig. 3a). Two out of six mice injected with SW480-NC cells had small lung metastatic nodules, whereas four out of six mice in the SW480-Gab2 group ended up with heavy lung metastasis. Conversely, silencing of Gab2 expression in SW620 cells had small lung metastatic nodules when compared with the control group. Moreover, the counts of lung and liver metastatic nodules exhibited significantly increased in the SW480-Gab2 group and significantly decreased in SW620-Gab2si group, compared with the corresponding control groups (Fig. 3b). These results suggest that Gab2 can enhance CRC cells metastasis in vivo.

### Gab2 regulates EMT in CRC cell

The EMT is a powerful process in tumor invasion and metastasis. During the EMT process, the molecular reprogramming and phenotypic changes characterized by a transition from polarized immotile epithelial cells to motile mesenchymal cells, thus leading to increased motility and invasion [17]. Moreover, the transition is characterized by a decrease in the expression of epithelial markers (such as E-cadherin) as well as an increase in the expression of mesenchymal markers (such as vimentin) [18, 19].

**Fig. 4:**
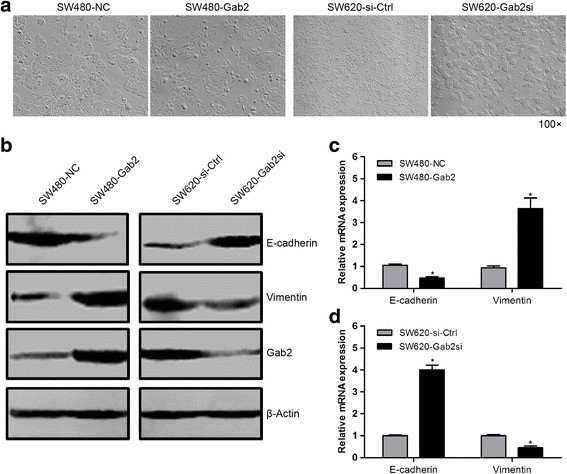
Gab2 regulates EMT in CRC cells. **a** Morphological changes by Gab2 in SW480 and SW620 cells. **b**, **c** and **d** Western blot and qRT-PCR show downregulated expression of E-cadherin and upregulated expression of vimentin in SW480-Gab2 cells. In contrast, silencing of Gab2 resulted in increased expression of E-cadherin and decreased expression of vimentin in SW620-Gab2si cells. *P < 0.05

By comparing the morphology of the cell models described above under a light microscope, we found that elevated expression of Gab2 in SW480 cells induced the conversion of polarized epithelial cells to spindle-shaped, fibroblast-like mesenchymal cells with decreased cell-cell contact. Conversely, silencing of Gab2 in SW620 cells exhibited an increase in cell-cell adhesion and an epithelioid morphology (Fig. 4a). To confirm that Gab2 induced EMT to promote CRC metastasis, we assessed the expression of EMT-markers in these cell models. As results, a decrease in the expression of E-cadherin and an increase in the expression of vimentin were observed in SW480-Gab2 cells, compared with the control cells (Fig. 4b, c). By contrast, a marked decrease in the expression of vimentin and a significant increase in the expression of E-cadherin were observed in SW620-Gab2si cells, compared with the control cells (Fig. 4b, d). These results suggest that Gab2 is critical for the acquisition of EMT characteristics and may contribute to the EMT-induced invasive phenotype in CRC cells.

### Gab2 positively regulates ERK signaling in CRC cell

**Fig. 5:**
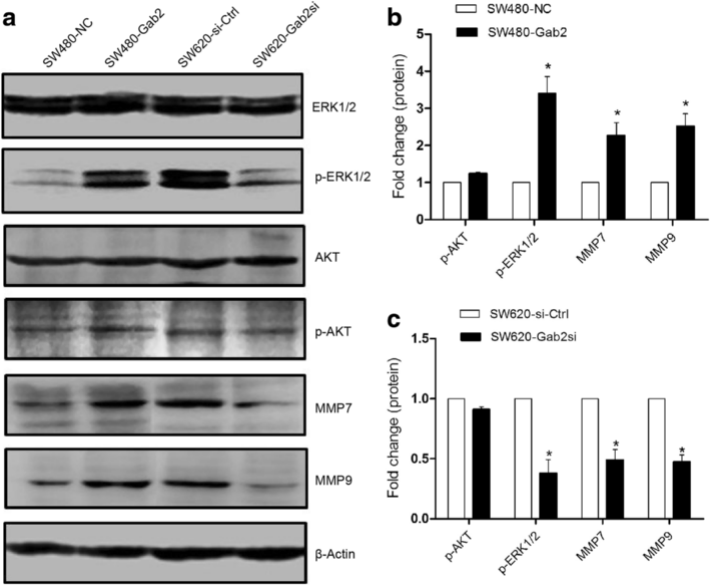
Gab2 expression regulates ERK signaling and increases MMP7 and MMP9 expression in CRC cells. **a** Gab2 overexpression enhances MMP7 and MMP9 expression and activates ERK1/2, whereas knockdown of Gab2 inhibits MMP7 and MMP9 expression and reduces ERK1/2 activation. **b** and **c** The levels of phosphor‑AKT, phosphor‑ERK1/2, MMP7 and MMP9 were calculated. The data are representative of at least three different experiments ± SEM. *P < 0.05

Recent studies have shown that the expression of several MMPs was enhanced in many types of cancer [20–22]. In these MMPs, it has been demonstrated that MMP7 and MMP9 were involved in the progression and metastasis of CRC [23–25]. To investigate the possible mechanism of Gab2 participating in cell metastasis of colorectal carcinoma, we examined its downstream effectors, AKT and ERK. Overexpression of Gab2 in SW480 cells significantly increased phosphorylation of ERK1/2, whereas knockdown of Gab2 in SW620 cells obviously reduced the activation of ERK1/2 (Fig. 5). Not as expected, the phosphorylation of AKT did not show marked increased or decreased in these cells (Fig. 5). Moreover, we also detected the expression of MMP7 and MMP9 in these cells, and found that increased MMP7 and MMP9 expression in SW480-Gab2 cells compared with the control cells, and decreased MMP7 and MMP9 expression in SW620-Gab2si cells compared with the control cells (Fig. 5). These data indicates that Gab2 may enhance CRC cells metastasis through the ERK/MMP pathway.

### Pharmacological inhibitor of MEK inhibits Gab2-induced EMT

**Fig. 6:**
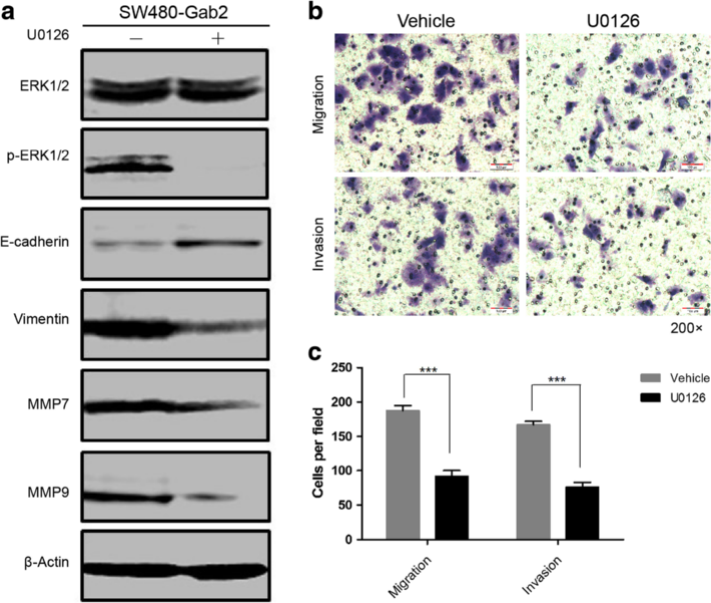
Inhibitor of MEK reduces Gab2-induced EMT, cell migration and invasion. **a** SW480-Gab2 cells were treated or not with 20 μM U0126 during 16 h after which proteins were analyzed by western blot with specific antibodies against E-cadherin, vimentin, MMP7, MMP9, phosphorylated ERK1/2 and total ERK1/2. **b** and **c** SW480-Gab2 cells were subjected to transwell migration and invasion assays in the absence (vehicle), or presence of 20 μM U0126. ***P < 0.001

To test the hypothesis that the commissioning of EMT by Gab2 is required for the activation of MEK/ERK signaling in colorectal carcinoma, we explore whether U0126, an effective MEK inhibitor, can inhibit Gab2-induced EMT and cell migration and invasion in CRC. Western blot analyses confirmed that the expression of E-cadherin was partially restored and the expression of vimentin, MMP7 and MMP9 was reduced upon using U0126 in SW480-Gab2 cells (Fig. 6a). Accordingly, migration and invasion of SW480-Gab2 cells were analyzed in the absence or presence of U0126 using transwell migration and invasion assays. As results, migration and invasion of SW480-Gab2 cells were significantly reduced in the presence of U0126 (Fig. 6b, c). These data indicates that Gab2-induced EMT and cell metastasis is dependent on MEK/ERK/MMP signaling in CRC.

## Discussion

It has been widely recognized that MEK/ERK signaling is participated in the progression and metastasis of CRC [26–28]. In addition, expression of a constitutively active mutant of MEK1 or MEK2 in rodent normal intestinal epithelial cells (IECs) is sufficient to induce growth, EMT and formation of invasive metastatic tumors in nude mice [29, 30]. In this study, we have shown that Gab2 promotes cell migration and invasion of CRC through activation of the MEK/ERK pathway, further showing the importance of ERK signaling for CRC metastasis. Additionally, we provide evidence supporting the involvement of Gab2 in the regulation of EMT in cancer.

Gab2 has recently been proposed to be a critical molecule in the regulation of cancer metastasis [10], although the exact mechanism of Gab2 in metastasis remains unclear. We found that overexpression of Gab2 enhanced CRC cell migration and invasive properties. Conversely, knockdown of Gab2 had the opposite effect. In accordance with our study, Gab2 was reported to be one of the molecules essential for both ovarian cancer and melanoma [12, 13]. Similarly, another study indicated that the level of Gab2 expression was significantly associated with metastatic progression of breast cancer [31]. Moreover, efficient ErbB2-driven mammary tumorigenesis and metastatic spread requires Gab2 expression [32]. Our results shed new light on the role of Gab2 in the promotion of cancer metastasis.

It has been reported that Gab2 regulates cytoskeletal organization and migration of mammary epithelial cells by modulating RhoA activation [33]. Interestingly, a recent study has shown that miR125a-5p could inhibit migration and invasion of glioma cells by mediating Gab2 to affect cytoskeleton rearrangement and MMPs expression [34]. These studies suggest that Gab2 might promote cancer metastasis by regulating the EMT. In this study, we demonstrated that upregulation of Gab2 expression induced the conversion of polarized epithelial cells to spindle-shaped, fibroblast-like mesenchymal cells with decreased cell-cell contact, enhanced invasion and migration in CRC cells, upregulated vimentin, MMP7 and MMP9 and downregulated E-cadherin. These findings provided new evidence supporting the involvement of Gab2 in driving cancer cells metastasis through the regulation of EMT.

Of further interest, we examined the possible pathway of Gab2 participating in cell metastasis of colorectal carcinoma and found that increased ERK1/2 phosphorylation in Gab2-upregulated CRC cells. Unfortunately, we did not detect any significant association between AKT phosphorylation and the expression of Gab2 in CRC cells. In addition, pharmacological inhibitor of MEK could reverse the effects of Gab2 on CRC cells migration and invasion, and partially restore E-cadherin expression, suggesting that Gab2-induced EMT is dependent on MEK/ERK signaling in CRC. Notably, activating genetic mutations in the PI3K/AKT and the MEK/ERK pathways have been implicated in CRC. Gab2 has been recognized as critical activators of the PI3K/AKT and/or the SHP2/ERK pathways in several cellular systems [10]. In theory, the level of Gab2 should affect AKT and ERK phosphorylation in CRC cells. However, SW620 cells transformed by downregulation of Gab2 did not show marked reduction of AKT phosphorylation in comparison with control cells. Similarly, at higher concentration ratios of PI3K/SHP2 in VEGF-stimulated endothelial cells, the lipid kinase is not limiting and AKT is phosphorylated to the same extent regardless of Gab2 knockdown, indicated that sufficient PI3K is activated for maximal AKT phosphorylation [35]. One plausible explanation is that the expression of Gab2 cannot markedly affect the concentration ratios of PI3K in these cells. Additionally, other Gab2-mediated oncogenic pathways need to be analyzed in order to clearly determine whether there is a link between Gab2 expression and EMT startup.

In summary, our study demonstrates that Gab2 could induce EMT and promote CRC cell metastasis through the MEK/ERK/MMP signaling. However, further studies are needed to pinpoint the target gene of Gab2, which can act as Gab2 promoters/suppressors regulated CRC metastasis. The present study provides a novel fundamental insight into how Gab2 promotes metastasis in colorectal carcinoma.

## Conclusions

Our study provides a better understanding on both the molecular mechanism and functional role of Gab2 in human CRC. Our current work revealed that Gab2 exerted its role as oncogene in CRC by facilitating cancer cell migration and invasion. Notably, Gab2 can upregulate MMP7 and MMP9 expression via MEK/ERK signaling pathway in CRC. Our study identified that Gab2 is a novel regulator of EMT through Gab2-medated MEK/ERK/MMP signaling, indicating its potential therapeutic value for reducing CRC metastasis.

## Additional file

Additional file 1: Table S1. Clinicopathologic factors and Gab2 expression in 35 CRC patients. (DOCX 22 kb)
