# Supplementary material for: Gab2 facilitates epithelial-to-mesenchymal transition via the MEK/ERK/MMP signaling in colorectal cancer
Source: J Exp Clin Cancer Res. 2016 Jan 12;35:5. doi: 10.1186/s13046-015-0280-0 (PMC4709914; doi:10.1186/s13046-015-0280-0)
Supplement: Additional file 1: Table S1. — Clinicopathologic factors and Gab2 expression in 35 CRC patients. (DOCX 22 kb) [file 13046_2015_280_MOESM1_ESM.docx]

| Characteristics | n(%) | | Gab2 expression | | *P* value | |
| --- | --- | --- | --- | --- | --- | --- |
|  |  |  | Low | High |  |  |
| Age (years) |  |  |  |  |  |  |
| ≥60 | 19(54.3) | | 8 | 11 |  |  |
| ＜60 | 16(45.7) | | 6 | 10 | 0.453 | |
| Gender |  |  |  |  |  |  |
| Male | 18(51.4) | | 7 | 11 |  |  |
| Female | 17(48.6) | | 7 | 10 | 0.808 | |
| Tumor size (cm) |  |  |  |  |  |  |
| ≥5 | 20(57.1) | | 7 | 13 |  |  |
| ＜5 | 15(42.9) | | 7 | 8 | 0.197 | |
| Lymph node status |  |  |  |  |  |  |
| No metastasis | 24(68.6) | | 12 | 12 |  |  |
| Metastasis | 11(31.4) | | 2 | 9 | 0.001 | |
| TNM stage |  |  |  |  |  |  |
| I-II | 23(65.7) | | 12 | 11 |  |  |
| III-IV | 12(34.3) | | 2 | 10 | 0.001 | |
